# A cross-species approach to identify transcriptional regulators exemplified for Dnajc22 and Hnf4a

**DOI:** 10.1038/s41598-017-04370-9

**Published:** 2017-06-22

**Authors:** A. C. Aschenbrenner, K. Bassler, M. Brondolin, L. Bonaguro, P. Carrera, K. Klee, T. Ulas, J. L. Schultze, M. Hoch

**Affiliations:** 1 Developmental Genetics & Molecular Physiology, Life & Medical Sciences Institute (LIMES), University of Bonn, Bonn, Germany; 2 Genomics and Immunoregulation, Life & Medical Sciences Institute (LIMES), University of Bonn, Bonn, Germany; 3 Single Cell Genomics and Epigenomics Unit at the German Center for Neurodegenerative Diseases and the University of Bonn, 53175 Bonn, Germany; 4 Department of Craniofacial Development and Stem Cell Biology, Dental Institute, King’s College London, SE1 9RT London, United Kingdom

## Abstract

There is an enormous need to make better use of the ever increasing wealth of publicly available genomic information and to utilize the tremendous progress in computational approaches in the life sciences. Transcriptional regulation of protein-coding genes is a major mechanism of controlling cellular functions. However, the myriad of transcription factors potentially controlling transcription of any given gene makes it often difficult to quickly identify the biological relevant transcription factors. Here, we report on the identification of Hnf4a as a major transcription factor of the so far unstudied DnaJ heat shock protein family (Hsp40) member C22 (Dnajc22). We propose an approach utilizing recent advances in computational biology and the wealth of publicly available genomic information guiding the identification of potential transcription factor candidates together with wet-lab experiments validating computational models. More specifically, the combined use of co-expression analyses based on self-organizing maps with sequence-based transcription factor binding prediction led to the identification of Hnf4a as the potential transcriptional regulator for Dnajc22 which was further corroborated using publicly available datasets on Hnf4a. Following this procedure, we determined its functional binding site in the murine Dnajc22 locus using ChIP-qPCR and luciferase assays and verified this regulatory loop in fruitfly, zebrafish, and humans.

## Introduction

Transcription is the first step of decoding the information in the genes of our genome. It is the basis for the proteins that the cells in our body can produce and shape their fate during development, their reaction to changes in their surroundings, or their contribution in pathogenesis. Regulation of gene expression is therefore a time-, cell type-, and stimulus-specific tightly controlled process.

Identifying regulating transcription factors (TFs) for a particular gene of interest (GOI) can reveal information about the context of that gene’s function as well as be useful in designing strategies to alter its expression level. Originally only addressed experimentally, computational approaches using large datasets have been introduced to identify TFs for GOIs in recent years^1^. Classical experimental approaches included cloning of site-specific genomic sequences, followed by intensive testing of individual sections of a genomic locus by reporter assays^1^. However, these assays only allowed the identification of functional sequences, but did not yet reveal the TF responsible for a specific gene regulation. Prior to computational prediction of TF binding motifs, TFs could only be postulated and assessed by electro mobility shift assays^2^, or chromatin immunoprecipitation followed by PCR or microarray (ChIP-PCR/ChIP-chip)^3^.

As a next step, experimentally determined binding sites for individual TFs were collected in databases such as JASPAR^4^ or Transfac^5^ and principles for motifs of a given TF were established computationally^6^. With whole genome information, such motifs were used to predict genome wide binding sites, although it also became quickly clear that this approach results in a large number of false positive results^7^. More recent tools improved this drawback considerably by including conservation information, p-value thresholds and by evaluating the quality of the motif^8^.

The advent of next generation sequencing again accelerated the development of technologies to study genomic regulation on a global level. RNA sequencing (RNA-seq) to assess global transcriptomes, the assessment of open chromatin by DNase-Seq^9^, NOMe-seq^10^ (Nucleosome Occupancy and Methylome sequencing) or ATAC-seq^11^ (Assay for Transposase-Accessible Chromatin using sequencing), and global TF binding maps by ChIP-seq^12^ allow the global assessment of TF expression, chromatin accessibility and the assessment of global binding maps for individual TFs. Consortia such as ENCODE (www.encodeproject.org) or IHEC (www.ihec-epigenomes.org) have compiled enormous publicly available datasets of global TF binding maps, which are now available for further mining. These approaches have broadened from a sole TF-GOI context to the attempt to compute complete TF networks^13^.

However, even in light of this very rich resource of available data, there are still limitations, when searching for TFs regulating a particular GOI. Moreover, it is probably not feasible to generate TF maps for any given tissue. While improving significantly during the last years, ChIP-seq data are still hampered by the required quality of the antibodies utilized for immunoprecipitation^14^ and for many TFs, ChIP-seq grade antibodies do not yet exist. Global transcriptome data provide information about expression levels of TFs; however, the mere expression level of TFs cannot necessarily predict its transcriptional activity. TFs are often subject to post-translational modifications and controlled by signalling cascades within the cell. As a consequence, experimental validation of the functionality of a particular TF at a given gene locus is a complementary requirement to the assessment of global binding maps when studying TF-GOI interactions. While many of the necessary steps were introduced individually, comprehensive workflows are not available. Moreover, many of these separate approaches are still tailored towards the domain expert, particularly computational biologists and bioinformaticians. Yet, linking different computational approaches is still a daunting task for a scientist focusing on the experimental assessment of transcriptional regulation. What is required is an easy-to-follow approach combining the existing genome-wide datasets, computational prediction and concise experimental validation of the TF-GOI interaction.

DnaJ heat shock protein family (Hsp40) member C22 (Dnajc22) is the vertebrate ortholog of *Drosophila melanogaster* Wurst – a gene, we previously identified as an essential factor for the functional development of the tracheal system – the respiratory organ of the fly^15^. It is conserved with single orthologs across the species^16^, yet there is neither functional data nor information about the regulation for its vertebrate counterparts.

Here, we show a step-by-step combined *in silico* and experimental approach of how to harness the wealth of already published transcriptomic and ChIP-seq data complemented with selective wet-lab validation to functionally identify a transcription factor for a particular gene of interest. Following this approach and using Dnajc22 as an example, we identified Hnf4a as a major transcriptional regulator.

## Results

### Identification of Hnf4a as a potential transcription factor for Dnajc22

**Figure 1:**
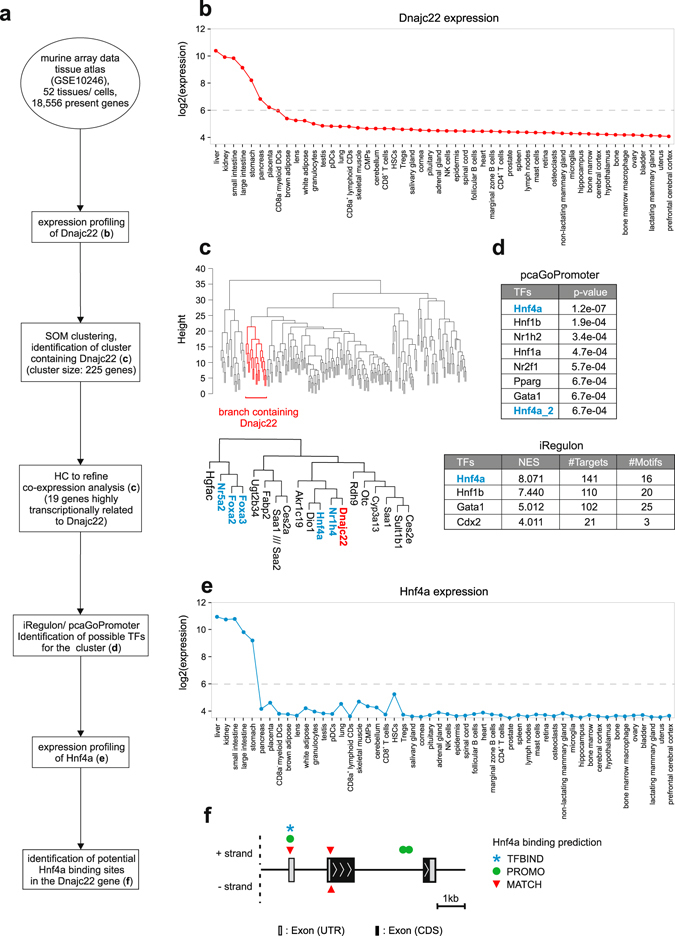
Identification of Hnf4a as a potential transcriptional regulator of Dnajc22. (**a**) Schematic workflow for the *in silico* analyses to identify potential transcriptional regulators of Dnajc22 expression (TF = transcription factor, HC = hierarchical clustering, SOM = self-organizing map). (**b**) Expression profile of murine Dnajc22. (**c**) Hierarchical clustering of genes which were grouped together according to self-organizing maps. Subcluster containing genes which were found to be highly transcriptionally related to Dnajc22 is highlighted in red. Transcription factors among those are marked in blue. (**d**) Potential TFs regulating genes contained in the Dnajc22-associated SOM-cluster identified by either iRegulon or pcaGoPromoter. TFs that are included in the SOM-cluster (**c**) themselves are marked in blue (TFs = transcription factors, NES = motif enrichment score). (**e**) Expression profile of Hnf4a. (**f**) Schematic representation of the murine Dnajc22 promoter region including possible Hnf4a binding sites predicted by TFBIND, PROMO, or MATCH. (UTR = untranslated region, CDS = coding sequence).

As a major principle for the identification of transcription factors (TFs) of any given gene of interest (GOI), we first postulated as a major requirement a substantial co-expression of TFs and the GOI across tissues and even species (Fig. 1a). As a model, we used the so far undescribed Dnajc22, the mammalian homologue of wurst, which we previously discovered to be involved in tracheal development in *Drosophila melanogaster* ^15^. According to the Ensembl database, wurst is evolutionary conserved, bearing single orthologs in at least 49 species including human and mouse^16, 17^ (Supplementary Fig. 1).

To determine co-expression of Dnajc22 with potentially regulating TFs in the mammalian system, we started with *Mus musculus*, for which large publicly available data sources concerning tissue, organ and cell-type specific gene expression are available. For this purpose, we utilized the murine gene atlas dataset (GSE10246)^18^ containing 182 transcriptomes of which we acquired 104 transcriptomes, pre-processed the data, and determined co-expression of genes across 22 organs, 14 tissues, and 16 cell types (Fig. 1a). The selection was based on covering the main tissues and cell types of the body as well as compiling an array of samples displaying the full expression extent of our GOI – including samples from anatomical locations expressing high levels of the GOI to such with none or very low levels. Visualization of expression values for Dnajc22 revealed a predominant expression in liver, kidneys, and intestine (Fig. 1b) while other closely related members of the J protein family (Dnajc3, Dnajc7, and Dnajc13)^19^ showed a different expression-profile (Supplementary Fig. 2a).

In order to identify potential TFs of our GOI, self-organizing map (SOM)-clustering was performed to determine those transcripts being co-expressed with Dnajc22 across organs, tissues, and individual cell types (Fig. 1c). The SOM-cluster containing Dnajc22 consisted of a total of 225 genes (listed in Supplementary Table 1), and hierarchical clustering of these genes revealed a Dnajc22-containing subcluster of 19 genes - five of which (Foxa2, Foxa3, Hnf4a, Nr1h4, Nr5a2) were TFs being co-expressed.

To identify potential transcription factors which could determine the expression of the 225 genes, two independent algorithms for predicting TF binding were used in an unbiased approach. iRegulon^8^ determined Hnf4a, Hnf1b, Gata1, and Cdx2, whereas the top results of pcaGoPromoter^20^ disclosed Hnf4a, Hnf1b, Nr1h2, as well as Hnf1a (Fig. 1d). Based on the hypothesis of co-expression for effective regulation, we overlaid these predictions with the transcripts contained in the SOM-cluster (Fig. 1c) and found Hnf4a to be closely co-expressed with Dnajc22 (Fig. 1e and Supplementary Fig. 2a). Additionally, we examined the transcriptional relationship between closely related family-members of Dnajc22 and all TFs which were predicted either by iRegulon or pcaGoPromoter by computing Pearson’s correlation coefficients and visualization of the results in a correlation coefficient matrix combined with hierarchical clustering (Supplementary Fig. 2b). One of the highest correlations was observed between Hnf4a and Dnajc22 confirming the co-expression results we have obtained before. We further substantiated these findings using three TF binding site prediction algorithms (TFBIND^21^, PROMO^22^, MATCH^23^), identifying five potential HNF4 binding sites within the murine Dnajc22 locus (Fig. 1f) indicating that Hnf4a may in fact be a prime TF candidate.

As there are several solutions for the initial identification of the set of co-expressed genes, we also tested a network approach based on weighted correlation network analysis (WGCNA)^24^. This alternative to our SOM-clustering approach shown in Fig. 1c also predicted Hnf4a as a potential regulator of Dnajc22 expression (Supplementary Fig. 3). Based on the phylogenetic conservation of Dnajc22 (Supplementary Fig. 1), we hypothesized a similar transcriptional regulation of the gene in other species. Therefore, we applied the same computational approach for the identification of transcription factors, as described for mouse samples above, also to human transcriptome data. The transcriptome data which we utilized for this purpose is part of a dataset published by the Roadmap Epigenomics Consortium^25^ (GSE16256) consisting of 25 transcriptomes composed of six organs, five tissues, and two cell types. As expected, SOM-clustering revealed a strong transcriptional relationship between DNAJC22 and HNF4A since both genes were found within the same cluster (Supplementary Fig. 4a). As for the murine data, iRegulon and pcaGoPromoter predicted HNF4A together with HNF1B and HNF4G as potential transcriptional regulators of SOM-cluster genes associated with DNAJC22 (Supplementary Fig. 4b). However, the transcriptional profile of DNAJC22 was strongest related to the tissue-specific expression of HNF4A (Supplementary Figs 4c and 5).

### Alteration of cellular Hnf4a levels affects Dnajc22 expression

**Figure 2:**
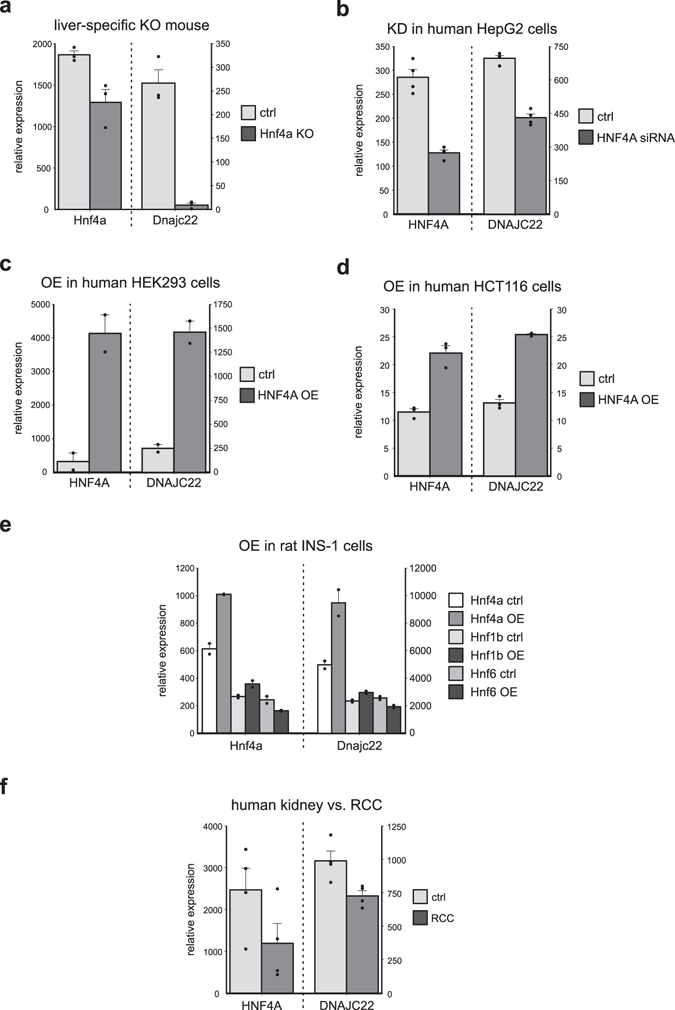
Alterations of cellular Hnf4a transcript levels affects Dnajc22 expression. Data for Hnf4a and Dnajc22 transcript levels in different Hnf4a-dependent experiments were extracted and reanalysed from online available GEO datasets. The significance of the observed differences was assessed using a one-sided t-test. (**a**) Hepatic tissue from liver-specific Hnf4a knockout and control mice (n = 3). (**b**) Knockdown of HNF4A by siRNA in human embryonic kidney cells (n = 4). (**c**) Doxycyclin-induced overexpression of HNF4A in human embryonic kidney cells (n = 2). (**d**) Doxycyclin-induced overexpression of HNF4A in human colon carcinoma (HCT116) cells (n = 3). (**e**) Doxycyclin-induced heterologous expression of HNF4A, HNF6, and HNF1B in rat insulinoma (INS-1) cells (n = 2). (**f**) Comparison of human healthy and renal carcinoma (RCC) tissue samples (n = 4).

As a next step, we searched for publicly available datasets in which loss- or gain-of-function experiments of Hnf4a were combined with whole transcriptome analysis (Fig. 2). We identified six studies in the GEO database^26–31^ and analysed the expression of Dnajc22 in these datasets. Microarray analysis of livers from a liver-specific Hnf4a knockout mouse model^26^ showed a reduction of Dnajc22 transcript to background levels (Fig. 2a). Knockdown of human HNF4A by siRNA in hepatocellular carcinoma cells^27^ also decreased DNAJC22 levels (Fig. 2b). Overexpression of HNF4A in human embryonic kidney cells^28^ as well as in human colorectal cancer cells^29^ induced DNAJC22 expression (Fig. 2c,d). Comparing the effect of HNF4A with that of HNF1B or HNF6 in rat insulinoma cells^30^ revealed that Dnajc22 levels were again influenced mainly by HNF4A with little contribution by HNF1B and no effect of HNF6 (Fig. 2e). Furthermore, HNF4A had been shown to be reduced in renal carcinoma samples^31^. Evaluating DNAJC22 levels from this study, we found DNAJC22 transcript to be diminished as well (Fig. 2f).

Making use of published transcriptome data, we identified several data sets of experiments that had altered cellular Hnf4a in either direction across several species. With the re-analysis of these data regarding Dnajc22, we provide further evidence that Hnf4a is a transcriptional regulator of Dnajc22.

### Hnf4a binds at the Dnajc22 locus

**Figure 3:**
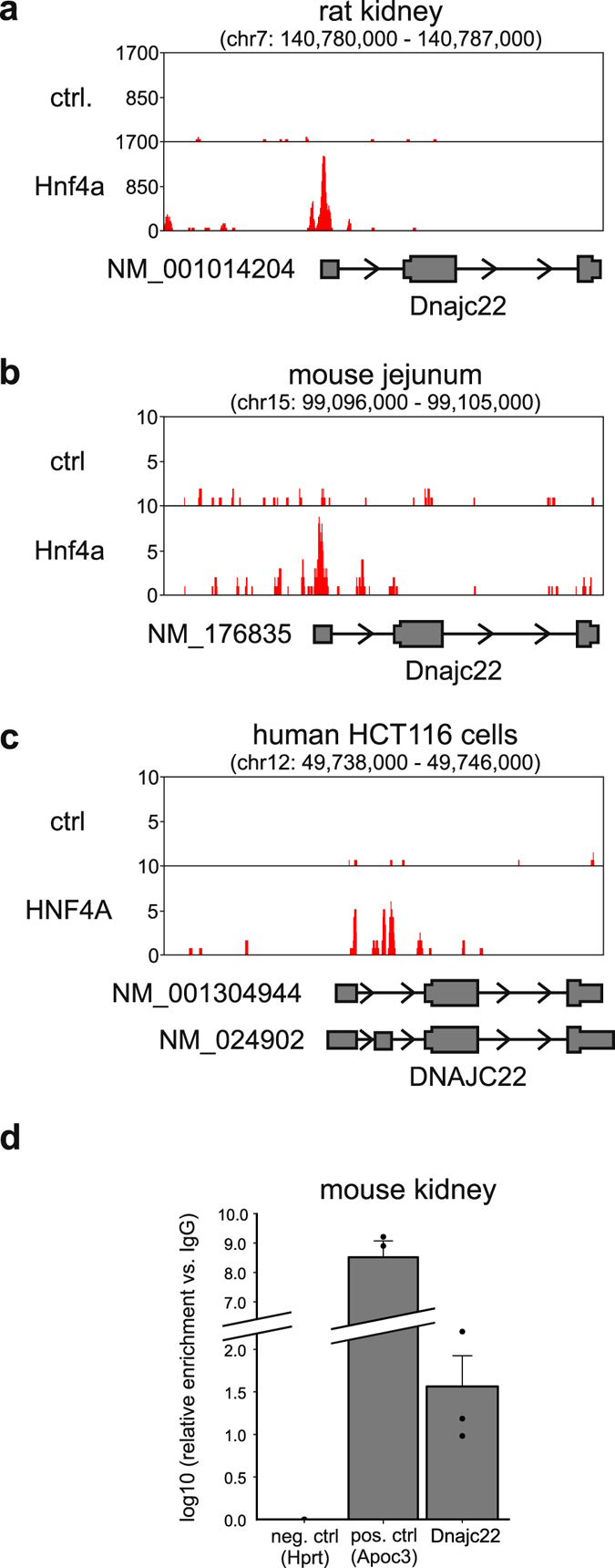
Hnf4a binds at the Dnajc22 locus. ChIP-seq peaks for Hnf4a at the rat Dnajc22 locus in kidney tissue (**a**), at the murine Dnajc22 locus in samples from intestine (**b**), and for HNF4A at the human DNAJC22 locus in human colon carcinoma (HCT116) cells (**c**). (**d**) HNF4A ChIP-qPCR of murine kidney cortex samples showing an enrichment of Hnf4a binding to the identified Dnajc22 promoter fragment. The Apoc3 promoter serves as a positive, a region in exon 9 of Hprt1 as a negative control. The significance of the enrichment was determined using a one-sided one-sample t-test.

An important step in the establishment of an actual regulatory connection between a TF and its target gene is finding proof of DNA binding at the target genomic locus. For this purpose, we searched for ChIP-seq experiments of Hnf4a in the GEO database, which resulted in three independent datasets from rat kidney^32^, mouse intestine^33^, and the human colon carcinoma cell line HCT116^29^. In the rat locus, two peaks were detected in the close vicinity of the Dnajc22 transcription start site by the peak-calling tool MACS2 (Fig. 3a). For murine (Fig. 3b) and human (Fig. 3c) DNAJC22 loci, ChIP-seq revealed one peak in the first or second exon, respectively, within the 5′ untranslated region of the gene. We further validated these findings by anti-HNF4A-ChIP-qPCR for the sequences from the first exon of the murine locus in murine kidney cortex samples (Fig. 3d). Collectively combining previous ChIP-seq data and our own ChIP-qPCR results we unequivocally demonstrate that Hnf4a is indeed a directly binding TF at the Dnajc22 locus in at least three species and two different organs.

### Identification of a functional Hnf4a binding site for murine Dnajc22

**Figure 4:**
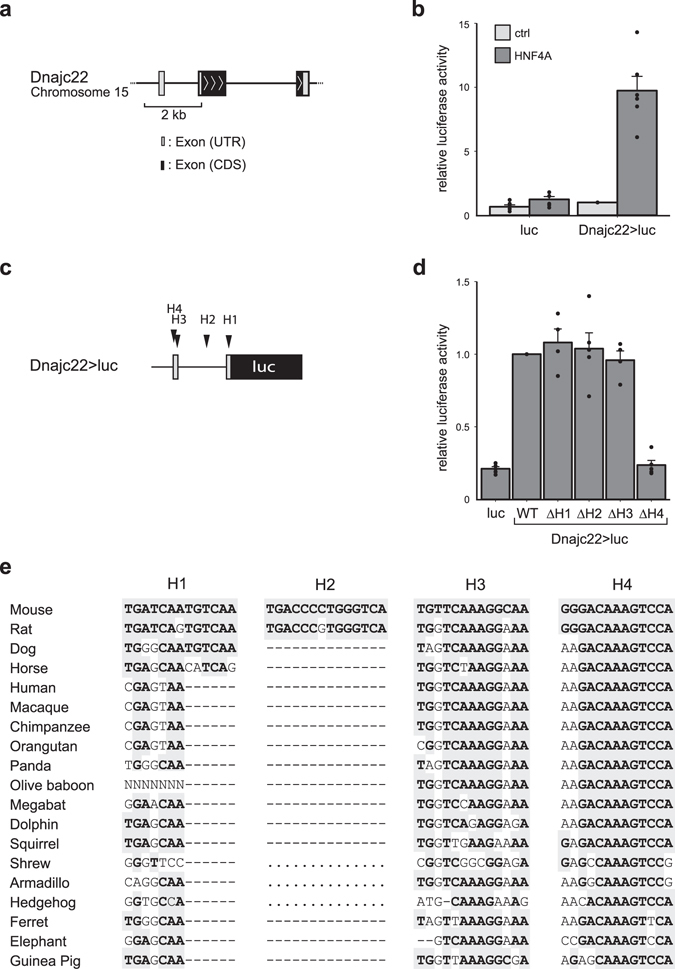
Identification of a functional Hnf4a binding site for murine Dnajc22. (**a**) Schematic presentation of the genomic locus of murine Dnajc22 indicating the 2 kb fragment cloned upstream of luciferase to test for responsiveness to heterologous HNF4A expression. (**b**) Heterologous expression of human HNF4A in murine M-1 cells increases luciferase activity of a construct driven by the 2 kb promoter fragment of murine Dnajc22 (normalized to Dnajc22 > luc control). Significance testing was performed using a one-sided paired t-test. (**c**) Predicted Hnf4a binding sites in the Dnajc22 > luc construct, denoted H1-4. (**d**) Analysis of single mutants of the four predicted Hnf4a binding sites (∆H1-4) show significantly reduced luciferase activity compared to the WT fragment after heterologous HNF4A expression only for ∆H4 (normalized to the WT construct). Significance testing was performed using a one-sided paired t-test. (**e**) Genomic alignment of the four identified potential HNF4A binding sequences in the Dnajc22 locus in 19 species including mouse, rat, and human shows greatest conservation for H4.

After demonstrating binding of Hnf4a to the Dnajc22 locus, we next sought to determine the functional consequence of TF binding by luciferase reporter assays. We cloned a 2 kb fragment of the murine *Dnajc22* promoter region upstream of a luciferase gene bearing only a minimal promoter for minimal background expression (Fig. 4a). The murine M-1 kidney cell line was chosen for its little Hnf4a expression to have a controllable system for studying the effect of this transcription factor. Heterologous expression of human HNF4A in these cells showed that the Dnajc22 promoter element was responsive to the TF as luciferase activity increased significantly (10.45 fold, P = 0.02, n = 6) compared to the control transfection (Fig. 4b). Utilizing the HNF4 Binding Site Scanner^34^, we identified four potential HNF4A binding sites (H1–H4) in the cloned sequence (Fig. 4c). Repeating the luciferase assay with versions of the original construct, in which each single predicted TF binding site was mutated, identified H4 at + 50 bp as functionally relevant. For H4, heterologous HNF4A expression showed luciferase activity at background levels (P = 0.0003, n = 4). Mutation of the other three potential sites did not reduce luciferase activity after heterologous HNF4A expression compared to the original construct (Fig. 4d). Importantly, the H4 motif had the highest conservation across species, further corroborating that H4 is the functionally most relevant binding site in the cloned promoter sequence of Dnajc22 (Fig. 4e).

### Experimental validation of Hnf4a-mediated regulation of Dnajc22

**Figure 5:**
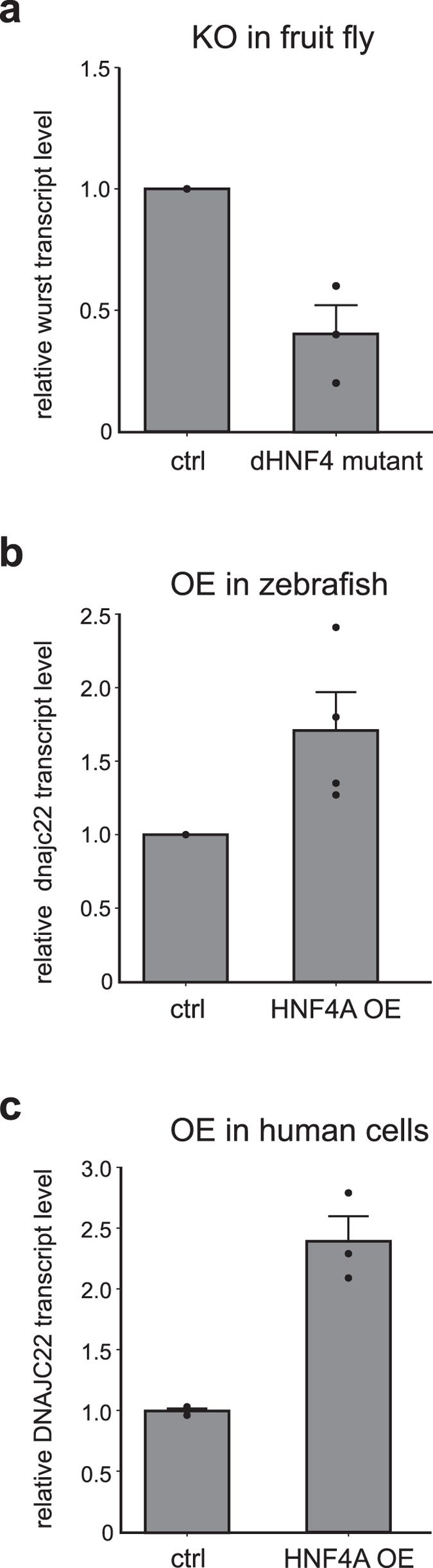
Hnf4a and Dnajc22 transcripts are co-regulated in fruitfly, zebrafish, and human. Endogenous expression of Dnajc22 ortholog transcript levels were analysed by quantitative realtime PCR in Hnf4a gain- and loss-of-function experiments. The data was generated from three independent experiments and normalized to the controls. Significance was tested using a one-sided paired t-test. (**a**) *Drosophila melanogaster* Dnajc22 ortholog wurst expression in Hnf4 mutant and control larvae. (**b**) Heterologous expression of human HNF4A in zebrafish embryos leads to elevation of zebrafish dnajc22 transcript levels. (**c**) Overexpression of human HNF4A in HEK293 cells leads to increased human DNAJC22 transcript levels.

Finally, we attempted to validate the Hnf4a-mediated regulation of Dnajc22 by loss- and gain-of-function experiments in additional model organisms including the fruitfly, zebrafish, and a human cell line. In the fruitfly, we used the previously described Hnf4 mutants^35^ to analyse the *Drosophila melanogaster* Dnajc22 ortholog wurst by qPCR demonstrating a similar co-regulation of these two genes (Fig. 5a) as we had observed in the previous transcriptome studies (Fig. 2). Heterologous expression of human HNF4A in zebrafish embryos induced the endogenous Dnajc22 transcript (Fig. 5b). DNAJC22 transcript levels were also elevated after transfecting human embryonic kidney cells with HNF4A (Fig. 5c).

## Discussion

The identification of upstream transcription factors (TFs) regulating a given gene of interest (GOI) is a major research area. Classical approaches including gene cloning strategies, reporter assays, and protein-DNA binding studies are labour-intensive, highly dependent on availability of respective reagents such as specific antibodies and often do not lead to the identification of the major transcriptional regulators. Here, we describe an integrated methodology that makes use of recent technological advances in genomics, transcriptomics, and epigenomics and is based on the hypothesis that TFs and their regulated GOIs are co-expressed across tissues and species. In a first step, applying clustering approaches to large transcriptome datasets such as human and murine tissue atlases, we identified co-expressed TF candidates for our GOI Dnajc22. Prediction for binding of these candidates to the Dnajc22 locus further decimated the number of true candidate TFs leaving Hnf4a as the most likely candidate regulating Dnajc22. An important next step was the *in silico* validation using additional publicly available transcriptome datasets, in which the TF was altered, e.g. in knockout, knockdown or overexpression experiments. Assessing the expression of the GOI in these datasets already strongly suggested that Dnajc22 is indeed directly regulated by the predicted TF. Direct binding of Hnf4a to the Dnajc22 locus was also evaluated *in silico* by re-assessing publicly available Hnf4a ChIP-seq datasets and further confirmed experimentally by ChIP-qPCR in kidney, which is one of the organs with the highest co-expression of both genes. Functional characterization of the binding was then performed by concise reporter assays for TF binding to the predicted binding sites. Together with *in silico* conservation analysis, we demonstrated that only the most highly conserved predicted binding site of Hnf4a in the Dnajc22 locus was of functional relevance. Finally, we proved the dependency of Dnajc22 orthologs on Hnf4a in species such as the fruitfly and zebrafish. Collectively, the wealth of publicly available genomic information enables us to utilize this information *in silico* for successful and less time-consuming identification of potential transcriptional regulators prior to further designing and performing more concise experiments to verify the hypothesis. Transcriptional regulators for Dnajc22 had previously not been reported. Following our strategy, we could identify Hnf4a as a transcription factor for Dnajc22 expression across several species.

As the primary input data source for co-expression analysis, we used the murine tissue array. Based on the assumption of similar regulation of Dnajc22 orthologs, an important step was also the confirmation of similar co-expression patterns between potential TFs and GOI in a second species, e.g. RNA-seq data from various human samples provided by the Roadmap Epigenomics Consortium (Supplementary Fig. 4). There are other resources that could also be included, e.g. tissue-specific resources such as the Human Brain Transcriptome (hbatlas.org) or resources focusing on certain systems like the immune system, e.g. the ImmGen dataset for murine immune cell types (www.immgen.org). Such specialized databases might be particularly interesting if a GOI is exclusively expressed in such a context. The availability of data sets in public repositories determines the general applicability of our proposed approach. Considering the exponentially growing numbers of novel transcriptome data published in recent years, the available opportunities in extending our approach to manifold initial datasets will expand even further.

As a first step of TF-identification, we used clustering algorithms to reduce the complexity of the used dataset. The initial co-expression analysis was a model-based clustering method, named self-organizing map (SOM)-clustering, which is among hierarchical clustering and K-means one of the most popular clustering methods in the field of gene expression analysis^36, 37^. A big drawback of both SOM and K-means is the need to predefine the number of clusters beforehand. However, SOM-clustering has been described to be more robust than other clustering methods when working with noisy data^38^. Furthermore, it is an excellent tool in exploring transcriptome data and has commonly been used in transcriptome analysis^39–41^. Although many additional clustering methods exist, all of them have their drawbacks and advantages and thus there is no one clustering method with the best performance for all clustering problems^42^. D’haeseleer and colleagues suggested using more than one clustering algorithm to overcome the limitations of a single clustering method^36^. Reanalysing the dataset used in Fig. 1 by utilizing a network approach based on weighted correlation network analysis (WGCNA)^24^ also predicted Hnf4a as potential regulator of Dnajc22 and hence confirmed the results obtained by SOM-clustering (Supplementary Fig. 3).

Global gene expression analyses, as well as the accumulating knowledge about chromatin structure and DNA accessibility, are currently advancing the algorithms for TF binding site predictions. In addition, the recent progress in optimizing TF prediction tools has considerably reduced the rate of false positive results. For example, iRegulon accesses large databases to combine information from several species and experiments to substantiate the predicted motifs and hence reduces the number of biologically less relevant motifs^8^. Other TF prediction tools such as pcaGoPromoter rely on smaller databases and thus are likely to produce less comprehensive results compared to e.g. iRegulon. However, in case of pcaGoPromoter, the great advantage is the implementation in R which e.g. permits easier integration in our R-based analysis workflow.

Overall, the strength of our approach lies in the combination of various data sources, approaches and tools, which allows us to focus on TFs fulfilling several criteria including co-expression of the TF in the subcluster of our GOI and binding prediction to the locus of our GOI. Although we focused on Hnf4a in the present study based on the high expression correlation to Dnajc22 (Supplementary Figs 2b and 5), we do not exclude additional effects of other transcription factors. Hnf1b for example was one of the factors that was also present in the murine and human Dnajc22-associated SOM-cluster and was found as potential regulator by the TF prediction tools. Hnf1b and Hnf4a have already been described as members of the same regulatory network, in which Hnf1b can induce Hnf4a^43^. However, in context of Dnajc22, Hnf1b did not show similar expression patterns across all cell types and tissues arguing against a global regulator for this gene. Collectively, although our presented example focussed on the prediction of the major transcription factor for our GOI, in principle, the approach can be utilized to find additional transcription factors for a GOI when using less stringent parameter thresholds.

Depending on the availability of transcriptomic and ChIP-seq data in the public domain, the resulting potential candidates can be further validated *in silico*. This is one of the major enhancements reducing experimental validation significantly as experimental data from gain- and loss-of-function experiments or even global TF-DNA-binding studies may already be available. The potentially wide array of available studies from various species, tissues and cell types analysed on different platforms or by differing methods (e.g. array vs. sequencing) will certainly show differences in the basal expression of the potential regulators or gene of interest, yet collectively enables to demonstrate concurrent changes in transcript levels. Once proteome data will be available to the same extent, one will be able to broaden the *in silico* validation step in our approach also on protein level.

**Figure 6:**
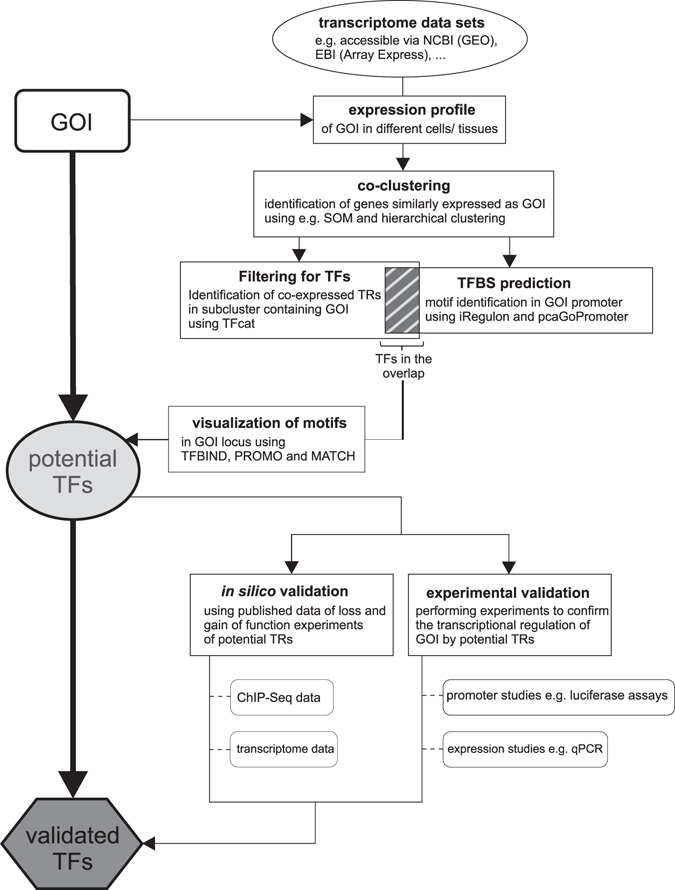
Schematic workflow. (GOI = gene of interest, SOM = self-organizing map, TFBS = transcription factor binding site, TR = transcriptional regulator).

Alongside discovering one of the major regulators of Dnajc22, we established a workflow that can be applied to any gene of interest. This overview is depicted in Fig. 6. A detailed, step-by-step version of this workflow with the data used in the present study can be found in Supplementary Fig. 7. Overall, starting with *in vivo* co-expression analysis of Dnajc22 with potential transcription factors, followed by *in silico* prediction of TF binding, evaluation of transcriptome data studying regulation of the potential TFs and ChIP-data addressing natural binding of the TF to the Dnajc22 locus, followed by functional analysis of the identified binding sites, and lastly experimental validation of regulation of Dnajc22 by the TF Hnf4a, we establish a straightforward workflow of identifying potential transcriptional regulators for any gene of interest. A major focus in designing the described strategy was that only freely available algorithms were used for analysis, opening the way for researchers to easily apply this strategy to their biological questions.

## Methods

### Use of publicly available information

This study makes use of publicly available data sets which were combined and analysed in a new context. These studies, gene accession numbers and names in different species as well as the utilized databases and tools are listed below.

*Drosophila melanogaster* wurst/CG9089/FBgn0030805

*Danio rerio* dnajc22/ENSDARG00000037067/si:dkey-166n8.8

*Rattus norvegicus* Dnajc22/ENSRNOG00000053498/RGD1311098

*Mus musculus* Dnajc22/ENSMUSG00000038009/2810451A06Rik

*Homo sapiens* DNAJC22/ENSG00000178401/FLJ13236

The phylogenetic tree for Dnajc22 was extracted from the Ensembl database^44^ from the Comparative Genomics section by choosing Gene Tree. GeneTree ID is ENSGT00390000012136.

The R-scripts used for analysing transcriptomes in this study are available on our GitHub page (https://github.com/LIMES-immunogenomics/Dnajc22-regulation).

### Microarray data pre-processing

Microarray gene expression dataset of murine tissue samples (GSE10246) was downloaded from NCBI GEO database. All pre-processing steps were performed using R version 3.3.0^45^. In detail, the dataset was loaded into R and subsequently normalized using the robust multi-array average (RMA) expression measure function of the Bioconductor package ‘affy’^46^. Genes were defined as expressed and kept for further analyses if the log2-transformed mean expression values were higher than the background value, which was set to 6. Afterwards, multi-probes were filtered to retain only a single probe with the highest variance across the dataset as representative for the corresponding gene. For subsequent clustering analysis, the mean gene expression values of the tissues were used. In the present study, 104 samples were used. However, we also performed the analysis with both all primary samples of the GSE10246 dataset (160 transcriptomes) and a reduced dataset (72 transcriptomes). Using these datasets and our provided workflow, we also identified Hnf4a as potential regulator of Dnajc22 expression (Supplemental Fig. S8).

### RNA-seq processing

Human RNA-seq samples from 6 organs, 5 tissues, and 2 cell-types were downloaded from NCBI GEO online database (GSE16256, specific SRR numbers see below) and converted from sra format to fastq using the fastq-dump function of sratoolkit 2.6.3 (https://github.com/ncbi/sra-tools). Reads were aligned to human reference genome hg19 from UCSC by HISAT2 version 2.0.4^47^ using default parameters. Quantification of aligned reads and extraction of gene and transcript information was performed using htseq-count (HTSeq version 0.6.1p1)^48^. Next, annotated data was imported into R and normalized using the Bioconductor package DESeq2^49^. For further analysis, only genes were used which exhibited a normalized RNA-seq group mean value higher than the 33^th^ percentile. Here, the cut-off value at the 33^th^ percentile was approximately 200; 16,141 genes had a group mean value higher than 200 and thus were kept for further analysis.

Organs:

spleen 1: SRR1220413 SRR1220414 SRR1220415 SRR1220416 SRR1220417 SRR1220418 SRR1220419 SRR1220420

spleen 2: SRR1220493 SRR1220494 SRR1220495 SRR1220496 SRR1220497 SRR1220498 SRR1220499 SRR1220500

small intestine 1: SRR651668 SRR651667 SRR651666 SRR651665

small intestine 2: SRR1220477 SRR1220478 SRR1220479 SRR1220480 SRR1220481 SRR1220482 SRR1220483 SRR1220484

sigmoid colon 1: SRR651669 SRR651670 SRR651671 SRR651672

sigmoid colon 2: SRR1220485 SRR1220486 SRR1220487 SRR1220488 SRR1220489 SRR1220490 SRR1220491 SRR1220492

pancreas 1: SRR651695 SRR651696 SRR651697 SRR651698

pancreas 2: SRR1220461 SRR1220462 SRR1220463 SRR1220464 SRR1220465 SRR1220466 SRR1220467 SRR1220468

lung 1: SRR577579 SRR577580 SRR577581 SRR577582

lung 2: SRR1220389 SRR1220390 SRR1220391 SRR1220392 SRR1220393 SRR1220394 SRR1220395 SRR1220396

liver: SRR651663 SRR651664

Tissues:

adipose 1: SRR1220373 SRR1220374 SRR1220375 SRR1220376 SRR1220377 SRR1220378 SRR1220379 SRR1220380

adipose 2: SRR1220445 SRR1220446 SRR1220447 SRR1220448 SRR1220449 SRR1220450 SRR1220451 SRR1220452

adrenal gland 1: SRR651679 SRR651680 SRR651681 SRR651682

adrenal gland 2: SRR1220421 SRR1220422 SRR1220423 SRR1220424 SRR1220425 SRR1220426 SRR1220427 SRR1220428

kidney cortex 1: SRR643771

kidney cortex 2: SRR643770

kidney pelvis 1: SRR643789

kidney pelvis 2: SRR643787

psoas muscle 1: SRR1220397 SRR1220398 SRR1220399 SRR1220400 SRR1220401 SRR1220402 SRR1220403 SRR1220404

psoas muscle 2: SRR1220469 SRR1220470 SRR1220471 SRR1220472 SRR1220473 SRR1220474 SRR1220475 SRR1220476

Cell-types:

CD4 T cells: SRR644513 SRR643766

CD8 T cells: SRR644512 SRR644514

### Self-organizing maps and hierarchical clustering

The complexity of the pre-processed transcriptome data was initially reduced by taking advantage of Kohonen’s self-organizing maps^50^. For this purpose, we utilized the ‘som’-function of the Bioconductor package ‘kohonen’^51^. Therefore, a training dataset was generated by randomly picking 1.000 genes from the transcriptome dataset. A tissue-wise z-transformation was computed on the basis of the training dataset and subsequently applied to the test dataset which was composed of the complete transcriptome data. Next, the self-organizing maps were trained using 100 iterations and a 10 × 10 grid. Finally, the trained model was applied to the test dataset. All genes which were predicted by SOM to be co-expressed with Dnajc22 were used as input for hierarchical clustering, which was performed using the ‘hclust’-function of R.

### Weighted correlation network analysis

To determine gene clusters associated with the 52 cell types/tissues of the mouse dataset, we have used the 18,556 present genes and applied the R implementation of the Weighted Gene Correlation Network Analysis (WGCNA^24^). We performed WGCNA clustering using the 1-TOMsimilarityFromExpr function, a power parameter of 6, a cut height of 0. 5, and a minimum module size of 20 dissecting the data into 29 modules.

### Co-expression network analysis

Pearson’s correlation was employed to compute the relationships between all gene pairs within the gene expression data. As input to this calculation, we used genes which were found in the same module like Dnjac22 (785 genes). Pearson’s correlation was calculated using BioLayout Express^3D^ ^52^.

Setting the correlation cut-off to 0.8 resulted in a network consisting of 464 nodes with 5,834 edges. Next, the gene-to-gene relationships were visualized in a force-directed layout in Cytoscape^53^.

### Transcription factors prediction using iRegulon and pcaGoPromoter

The Cytoscape^53^ plug-in iRegulon^8^ was used to predict TFs potentially regulating the expression of genes which were predicted to be co-expressed to Dnajc22. The genomic region for TF-prediction was limited to 10kbp centred around the respective transcriptional start sites. The statistical threshold settings were left unaltered. We confirmed the robustness of the iRegulon results against parameter changes (Supplementary Fig. 6).

In addition to iRegulon, we also utilized the default settings of the PRIMO tool which is implemented in the Bioconductor package ‘pcaGoPromoter’ for TF-prediction^20^.

### Pearson correlation coefficient matrix

To investigate gene-to-gene relationships, we utilized the ‘cor’-function of R to compute the Pearson’s correlation between a pair of genes based on transcriptome data. The calculated correlations were subsequently visualized in a heatmap resulting in a correlation coefficient matrix.

### Identification of Hnf4a binding motifs in the Dnajc22 gene using TFBIND, PROMO and MATCH

To screen the sequence of the Dnajc22 gene for potential Hnf4a binding sites, we took advantage of three different TF binding site prediction tools. As input we used the genomic sequence of the Dnajc22 locus with an extension of 1 kb up- and down-stream of the first and last exon respectively. In the present study, the default settings of the respective tools were adjusted as followed. TFBIND^21^ settings were adjusted to a similarity cut-off of 0.85. The PROMO^22^ algorithm was limited solely to HNF4A-motifs and the dissimilarity cut-off was set to 5%. MATCH^23^ was used with its default settings.

### Reanalysis of publicly available gene expression studies

Values for Hnf4a and Dnajc22 transcript levels were retrieved from the following studies available in the NCBI GEO database: GSE3126, GSE29084, GSE2700, GSE62891, GSE1589, GSE781 via the GEO2R profile graph option.

### ChIP-seq processing

ChIP-seq samples from human (GSE62890: SRR1636061, SRR1636082), mouse (GSE35568: SRR391524, SRR391525), and rat (GSE50815: SRR980345, SRR980344) were downloaded from NCBI GEO online database and converted from sra format to fastq using the fastq-dump function of sratoolkit 2.6.3. Reads from all three species were aligned to their reference genomes (hg19, mm10 and rn6 from UCSC) by Bowtie1 version v1.1.1^54^ using best match parameters (bowtie -t -q -e 70 -l 28 -n 2 –best–maxbts 125 -S). Peaks between control and treatment samples were called by MACS2 (version 2.1.0.20140616) using default parameters. Visualization of ChIP-seq peaks was performed with the Integrative Genomics Viewer (IGV)^55^.

### Chromatin immunoprecipitation-qPCR

Murine renal cortex tissue was cut into small pieces, digested with 0.05% trypsin and 1 mg/ml collagenase for 1 h at 37 °C, fixed with 1% formaldehyde and quenched with 125 mM glycine. Cells were resuspended in sonication buffer including protease inhibitors. Cell lysis and chromatin fragmentation were performed in two subsequent steps using a Covaris sonicator. Chromatin/protein complexes were incubated with anti-HNF4A antibody (sc-374229, Santa Cruz) to capture endogenous Hnf4a or mouse IgG (Sc2025, Santa Cruz) as a control, followed by addition of protein G-conjugated magnetic beads (Life Technologies). Chromatin/protein crosslinking was reversed and the DNA was purified with the NucleoSpin Gel and PCR Clean-up kit (Macherey Nagel). Binding enrichment was quantified by real-time PCR using the Apoc3 promoter as a positive and a Hprt1 coding region as a negative control^26^.

### Luciferase reporter assays

A 2 kb fragment of the murine Dnajc22 locus was subcloned into pGL4.24 (Promega/Dnajc22>luc). Potential HNF4a binding sites in the cloned sequence were identified using the Support Vector Machine (SVM) of the HNF4 Binding Site Scanner (http://nrmotif.ucr.edu)^34^. Various sites were mutated using the Q5 Site-Directed Mutagenesis Kit (NEB) following the manufacturer’s instructions.

M-1 cells (ECACC #95092201) were maintained following ECACC recommendations. Co-transfections were performed with Lipofectamine2000 (Life Technologies) containing Renilla luciferase bearing pGL4.74 (Promega) for normalization of transfection efficiency, empty pGL4.24 or Dnajc22 > luc as well as HNF4A or an empty control vector. Luciferase activity was assessed 22–25 h after transfection using the Dual-Glo Luciferase Assay System (Promega).

### H1-4 sequence alignment

Alignment of the four identified potential HNF4A binding sites was extracted from the genomic alignments in the comparative genomics section on the Ensembl page of murine Dnajc22 (based on Ensembl release 86, Oct 2016)^17^.

### Expression studies by quantitative real-time PCR analysis

Hnf4 mutant flies were a kind gift of Carls S. Thummel (Dep. of Human Genetics, University of Utah, USA). Transheterozygous crossings for precise excisions of the EP2449 and KG08976 P-elements, and Hnf4Δ33/Hnf4Δ17 deletion alleles were established as described^35^ and RNA was extracted from L2 larvae.

For heterologous expression studies in zebrafish, human HNF4A (FR_HNF4As, Addgene plasmid #31100)^30^ was subcloned into pCS2+ and *in vitro* transcribed (mMESSAGE mMACHINE SP6 Transcription kit, Ambion). Zebrafish embryos were obtained from WT TL crossings and injected with 25 ng HNF4A mRNA each. Samples were processed for RNA extraction after 24 h.

HEK293 cells (ATCC # CRL-1573, kind gift of Thomas Magin, Division of Cell & Developmental Biology, University of Leipzig, Germany) were cultured in DMEM (high glucose) including 10% FCS, GlutaMAX and penicillin/streptomycin (Life Technologies) at 37 °C/5% CO_2_. Human HNF4A or empty control vector were transfected using Lipofectamine2000 (Life Technologies) and RNA was prepared after 24 h.

RNA was isolated (RNA II, Macherey-Nagel), cDNA synthetized (QuantiTect, Qiagen), and quantitative real-time PCR was performed using the iQ SYBR Green Supermix (Bio-Rad). Expression was normalized to rp49 and actin (*Drosophila melanogaster*), eif1a and rpl13 (*Danio rerio*) and Hprt (*Mus musculus and Homo sapiens*).

### Statistical analysis

Where applicable, results are presented as mean ± standard deviation, together with individual data points to depict the data range. Normality of residuals was confirmed with Shapiro-Wilk tests (p > 0.05). Differences were assessed with t-tests and considered significant when p-value was p < 0.05. Marginal significance, also referred to as trends, was defined as p < 0.1.

## Electronic supplementary material

Supplementary Files

Supplementary Table 1
