# Supplementary material for: A cross-species approach to identify transcriptional regulators exemplified for Dnajc22 and Hnf4a
Source: Sci Rep. 2017 Jun 22;7:4056. doi: 10.1038/s41598-017-04370-9 (PMC5481429; doi:10.1038/s41598-017-04370-9)
Supplement: Supplementary file 1 — Supplementary Files [file 41598_2017_4370_MOESM1_ESM.pdf]

Supplementary Information

A cross-species approach to identify transcriptional regulators exemplified for Dnajc22 and Hnf4a

Aschenbrenner AC, Bassler K, Brondolin M, Bonaguro L, Carrera P, Klee K, Ulas T, Schultze JL, Hoch M

Supplementary Figure 1: Ensembl GeneTree for Dnajc22

Supplementary Figure 2: Comparison of expression of Dnajc22 and candidate transcription factors

Supplementary Figure 3: WGCNA-based approach for identification of TFs regulationg Dnajc22 expression

Supplementary Figure 4: Identification of HNF4A a potential transcriptional factor of DNAJC22

Supplementary Figure 5: Pearson's correlation matrix of human genes

Supplementary Figure 6: Effect of different parameter settings on iRegulon results

Supplementary Figure 7: Step-by-step workflow of the present study

Supplementary Figure 8: SOM-clustering of murine data using different sample sizes

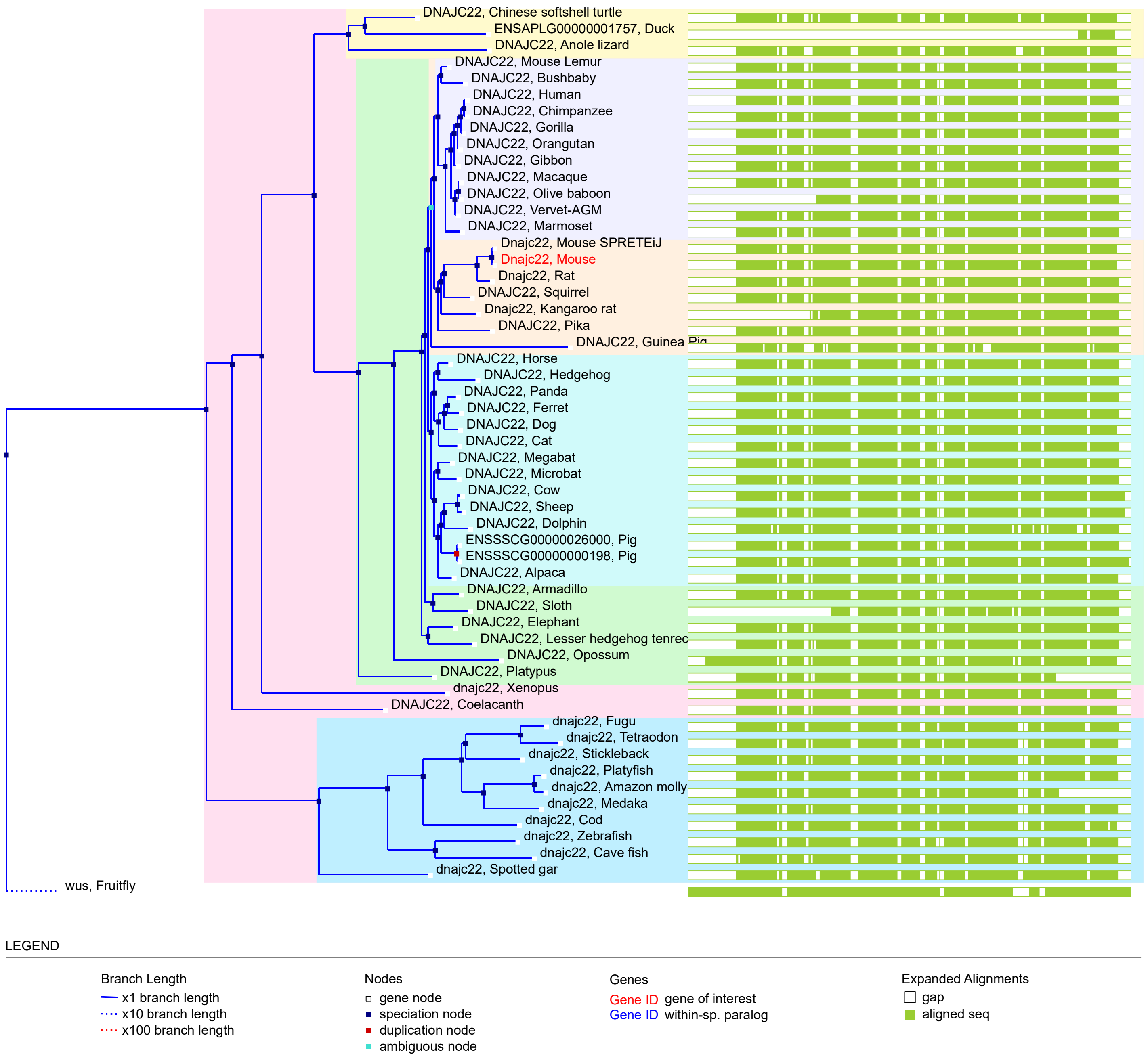

Supplementary Figure 1 - Ensembl GeneTree for Dnajc22

Maximum likelihood phyologenetic tree (Yates, A. et al. Nucleic Acid Res. 2016) based on murine Dnajc22 (red). Internal nodes are marked in blue for speciation or red for duplication events. Protein alignment next to the tree is depicted in green. The white areas show gaps in the alignment.

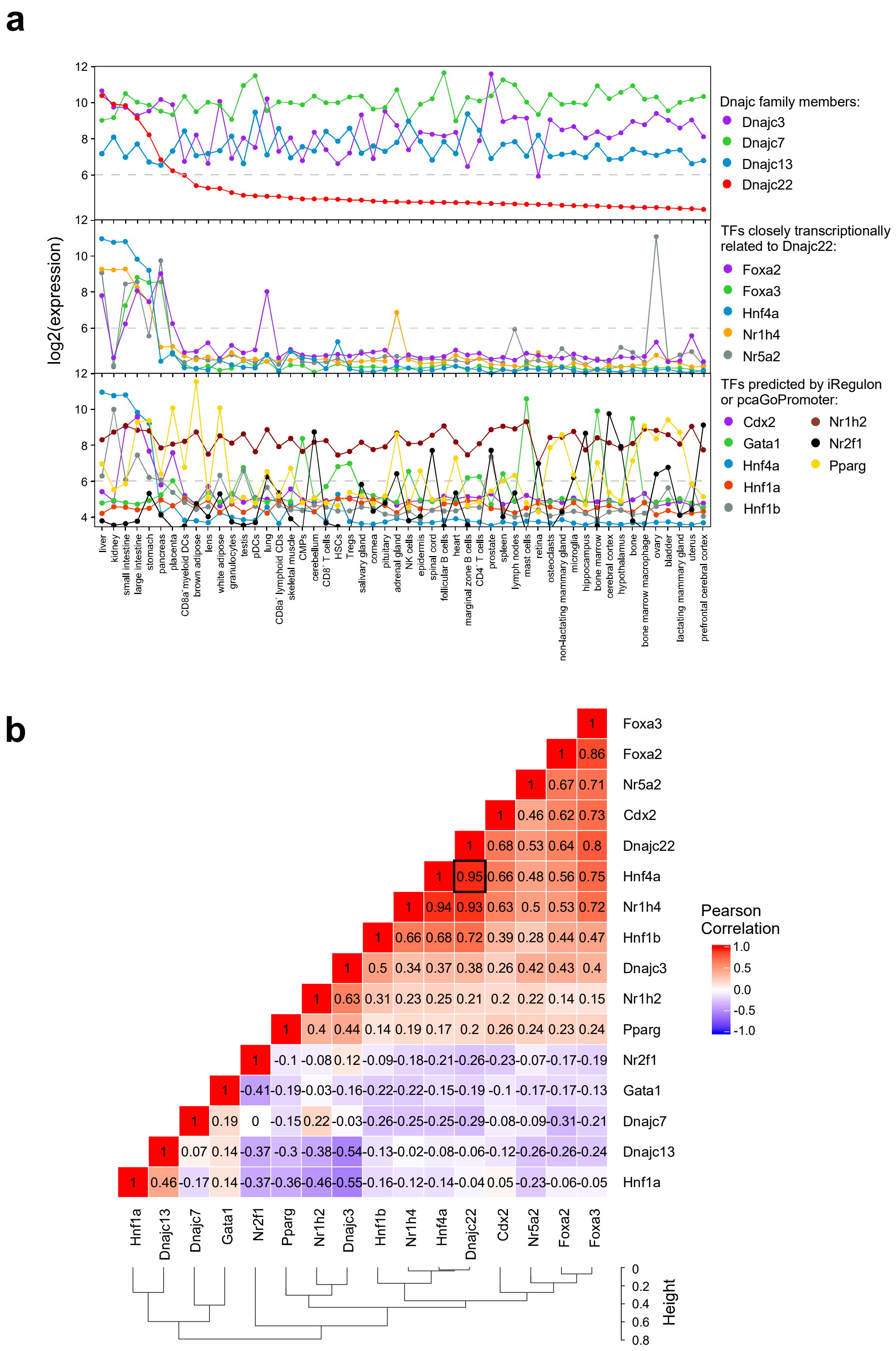

Supplementary Figure 2 - Comparison of expression of Dnajc22 and candidate transcription factors

a Tissue expression profiles for the Dnajc22-related family members Dnajc3, Dnajc7, Dnajc13 (first panel), transcription factors identified in the Dnajc22-containing subcluster (Fig. 1c / second panel here), and those predicted by iRegulon and pcaGoPromoter (Fig. 1d / bottom panel here).

b Pearson's correlation matrix for Dnajc22, Hnf4a, Dnajc22-related family members, as well as all identified potential transcription factors shown in **a**.

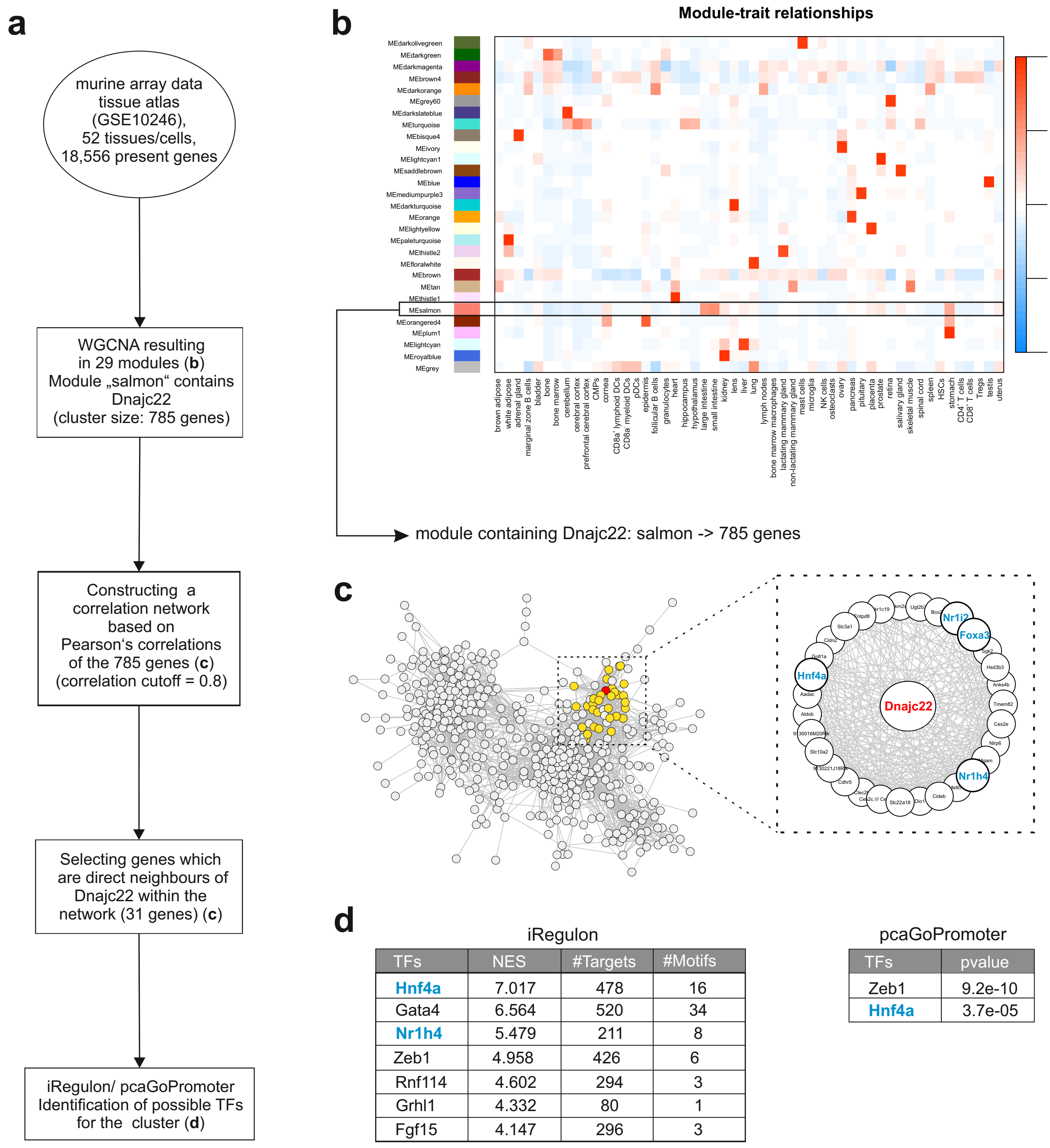

TFs	NES	#Targets	#Motifs
**Hnf4a**	7.017	478	16
Gata4	6.564	520	34
**Nr1h4**	5.479	211	8
Zeb1	4.958	426	6
Rnf114	4.602	294	3
Grhl1	4.332	80	1
Fgf15	4.147	296	3

TFs	pvalue
Zeb1	9.2e-10
**Hnf4a**	3.7e-05

Supplementary Figure 3 - WGCNA-based approach for identification of TFs regulating Dnajc22 expression

a Schematic workflow (TF = transcription factor, WGCNA = weighted correlation network analysis)

b WGCNA results represented in a heatmap showing the correlation of the module eigengenes (first principal component of the respective module) to the tissues/cells. Negative correlation depicted in blue, positive correlation in red.

c Correlation network of genes found in the same WGCNA module like Dnajc22 and sub-network constructed using Dnajc22 (marked in red) and its direct neighbours (marked in yellow). TFs within the sub-network are marked in blue.

d Possible TFs for the genes contained in the WGCNA module identified by either iRegulon or pcaGoPromoter. TFs that are found to be directly connected with Dnajc22 in the correlation sub-network (c) are marked in blue.

a

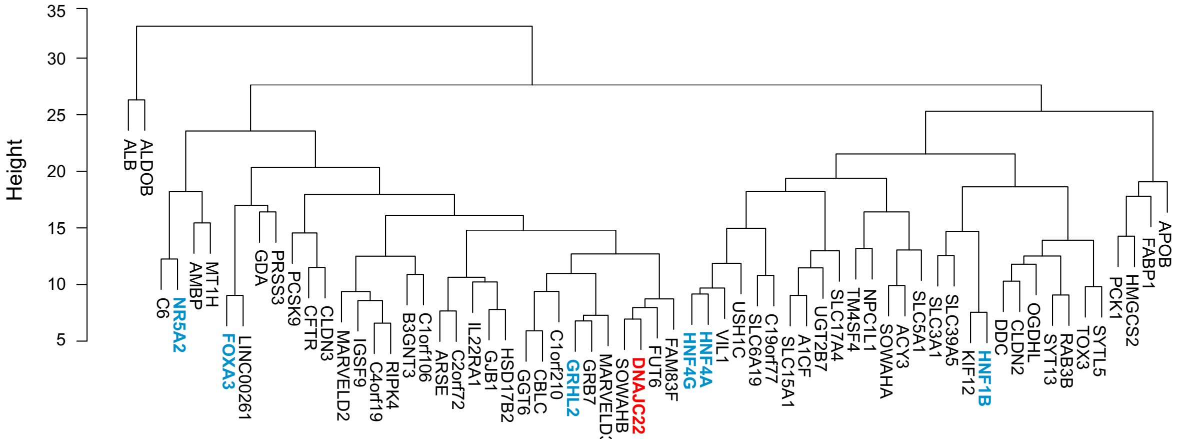

b

iRegulon

TFs	NES	#Targets	#Motifs	#Tracks
**HNF1B**	9.827	52	29	
**HNF4A**	8.980	44		3
EP300	6.207	35		4
ZEB1	5.637	19		2
NFIC	5.348	9		1
**HNF4A**	5.243	48	21	
FOXA1	4.991	35		3
**HNF4G**	4.795	37		2
HDAC2	4.751	18		1
JAZF1	4.666	31	7	
EVI1	4.660	22		1
NKX1-2	4.463	17	7	
CRX	4.148	23	2	
MBD4	4.006	6		1

pcaGoPromoter

TFs	pvalue
**HNF4A**	0.0004
PBX1	0.005

c

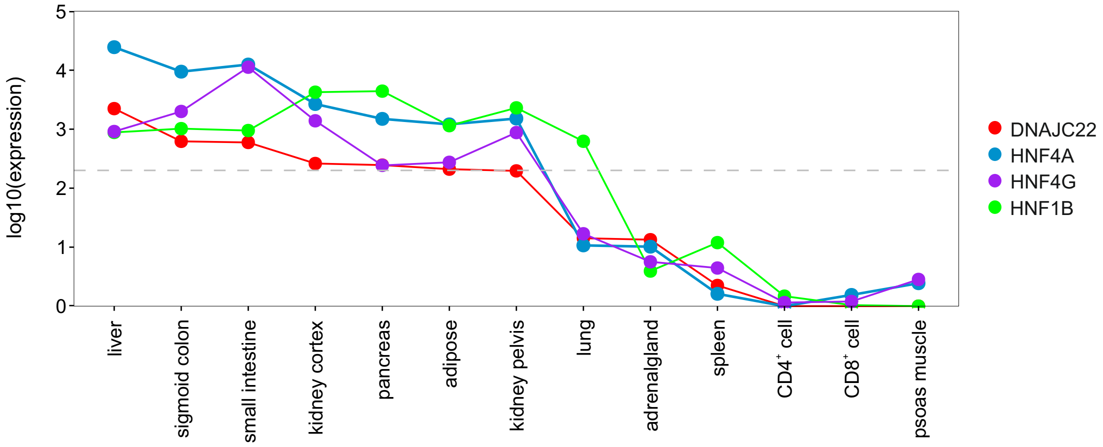

Supplementary Figure 4 - Identification of HNF4A as a potential transcriptional factor of DNAJC22

a Hierarchical SOM-cluster for 64 genes co-regulated with human DNAJC22 (highlighted in red). Transcription factors identified in the gene of interest-subcluster are marked in blue.

b Possible TFs for the genes contained in the SOM-cluster predicted by either iRegulon or pcaGoPromoter. TFs that are included in the SOM-cluster (**a**) are marked in blue (TFs = transcription factors, NES = motif enrichment score, SOM = self-organizing map); Tracks = hits based on ChIP-Seq datasets.

c Tissue expression profiles of DNJAC22, HNF4A, HNF4G, and HNF1B.

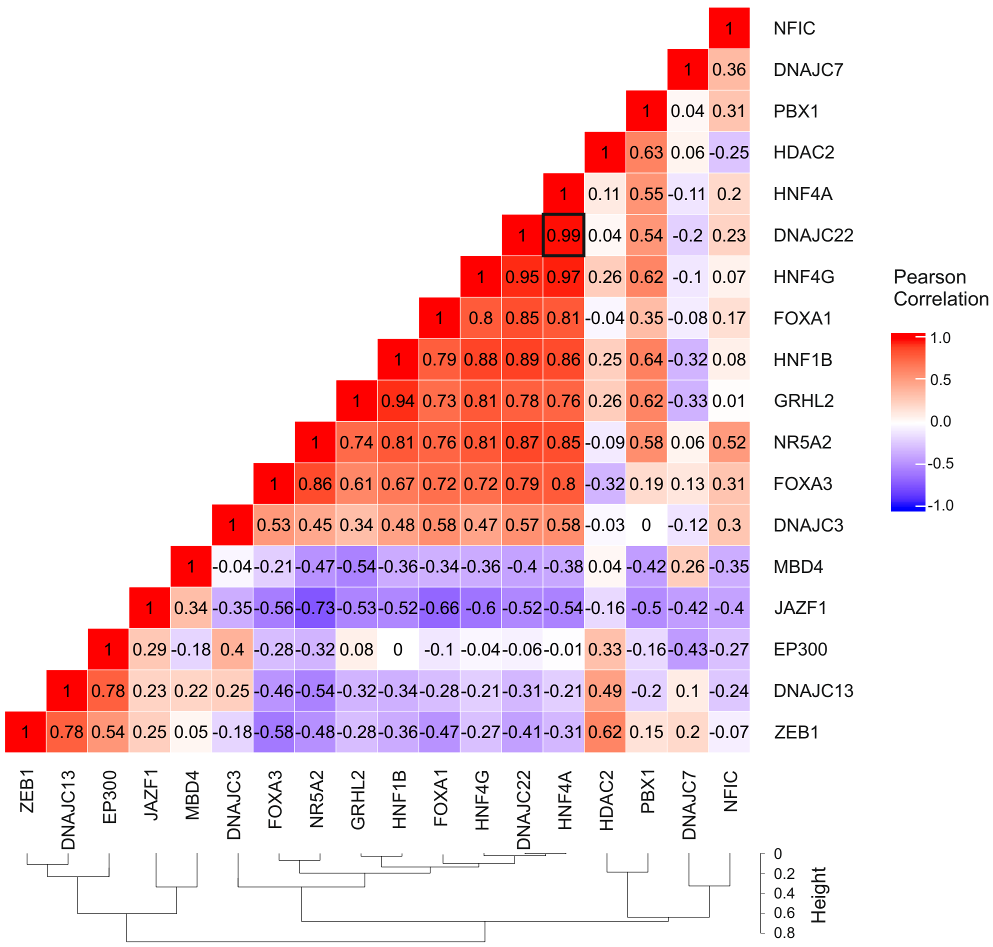

Supplementary Figure 5 - Pearson‘s correlation matrix for DNAJC22, HNF4A, DNAJC22-related family members, as well as all identified potential transcription factors shown in **Supplementary Figure 4b.** (TF = transcription factor)

Parameters (as used in Fig. 1)
Motif collection: 9713 PWMs
Putative reg. region: 10kb cent. around TSS
NES cutoff: 4
ROC threshold for AUC calculation: 0.03

TFs	NES	#Targets	#Motifs
Hnf4a	8.071	141	16
Hnf1b	7.440	110	20
Gata1	5.012	102	25
Cdx2	4.011	21	3

Parameters
Motif collection: 6383 PWMs
Putative reg. region: 10kb cent. around TSS
NES cutoff: 4
ROC threshold for AUC calculation: 0.03

TFs	NES	#Targets	#Motifs
Hnf1b	5.960	83	7
Hnf4a	4.837	118	7
Gata5	4.482	107	24

Parameters
Motif collection: 9713 PWMs
Putative reg. region: 500bp upstream of TSS
NES cutoff: 4
ROC threshold for AUC calculation: 0.03

TFs	NES	#Targets	#Motifs
Hnf4a	10.517	140	37
Hnf1b	7.295	85	33
Gata6	5.585	105	53

Parameters
Motif collection: 9713 PWMs
Putative reg. region: 20kb cent. around TSS
NES cutoff: 4
ROC threshold for AUC calculation: 0.03

TFs	NES	#Targets	#Motifs
Hnf1b	8.250	84	17
Hnf4a	6.375	105	12
Mecom	4.840	63	14
Nr1h4	4.180	87	7

Parameters
Motif collection: 9713 PWMs
Putative reg. region: 10kb cent. around TSS
NES cutoff: 4
ROC threshold for AUC calculation: 0.05

TFs	NES	#Targets	#Motifs
Hnf1b	7.710	113	20
Hnf4a	7.307	152	17
Gata1	4.505	98	21

Parameters
Motif collection: 9713 PWMs
Putative reg. region: 10kb cent. around TSS
NES cutoff: 4
ROC threshold for AUC calculation: 0.01

TFs	NES	#Targets	#Motifs
Hnf4a	7.650	137	13
Hnf1b	6.450	106	16
Gata1	4.894	103	28
Mecom	4.575	47	4
Luzp	4.452	8	1
2310045N01Rik	4.029	16	4

Supplementary Figure 6 - Effect of different parameter settings on iRegulon results

Changes of the parameter settings compared to those used in Figure 1 are highlighted in yellow.

(PWM = position weight matrix, TSS = transcription start site, NES = normalized enrichment score, ROC = receiver operating characteristic, AUC = area under the cumulative recovery curve, TF = transcription factor)

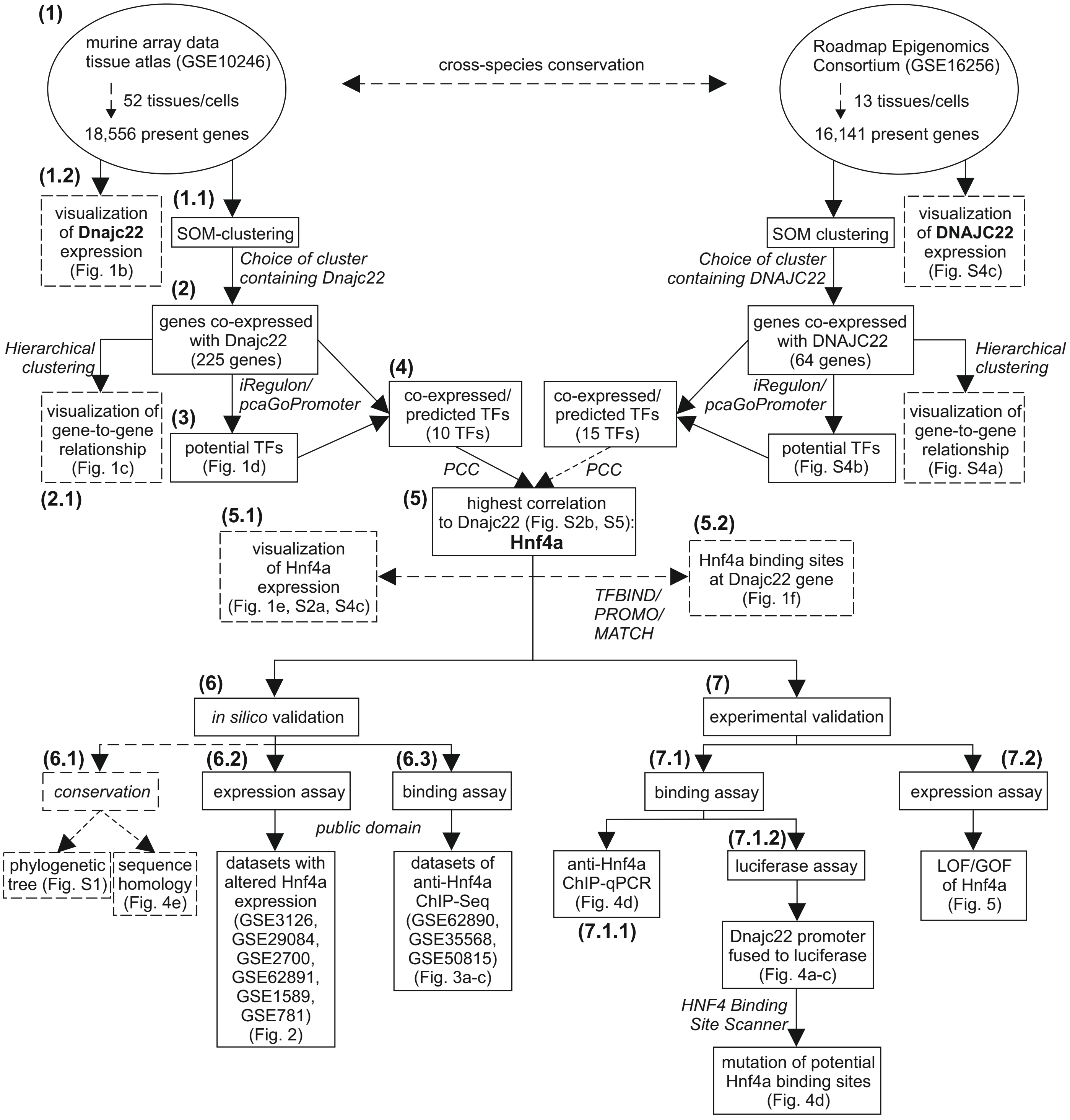

Supplementary Figure 7 - Step-by-step workflow of the present study

(SOM = self-organizing map, TF = transcription factor, PCC = Pearson‘s correlation coefficient analysis, ChIP-Seq = chromatin immunoprecipitation followed by DNA sequencing, ChIP-qPCR = chromatin immunoprecipitation followed by quantitative real-time PCR, LOF = loss of function , GOF = gain of function)

General step-by-step protocol

The presented workflow is based on an R script which is deposited on our GitHub page (https://github.com/LIMES-immunogenomics/Dnajc22-regulation).

1. Screen databases in public domain for transcriptome data
In the present study, 104 samples of the murine tissue atlas (GSE10246) were used. After downloading of the CEL files, the samples were RMA normalized followed by removing genes with a group mean expression < 6. Afterwards, multi-probes were filtered to retain only a single probe with the highest variance in the whole dataset as representative for the corresponding gene. Finally, 18,556 unique present genes, which represent the most informative genes, were kept for further analysis (section in the R-based workflow: "Data pre-processing").

1.1. Perform SOM-clustering on present genes (section in the R-based workflow: "SOM-clustering").

1.2. Visualization of expression of the gene of interest (GOI) across cells/tissues by using ggplot-function provided in the R-based workflow (section "Expression profile")

2. Selecting SOM-cluster containing the GOI

2.1. Visualization of genes within the GOI-associated SOM-cluster using hierarchical clustering

3. Prediction of potential transcription factors (TFs) regulating the expression of genes within the GOI-associated SOM-cluster by using either the PRIMO implementation of pcaGoPromoter (section in the R-based workflow: "TF prediction") or the Cytoscape plugin iRegulon

4. Making a list of TFs which were either found to be highly co-expressed to the GOI or which were predicted as a potential regulator of genes within the GOI-associated SOM-cluster. Compute a Pearson's correlation coefficient matrix (section in the R-based workflow: "Pearson's Correlation Coefficient Matrix")

5. Selecting TF with the highest correlation to the GOI

5.1. Visualization of that TF's expression across cells/tissues by using ggplot-function provided in the R-based workflow (section "Expression profile")

5.2. Examination of potential binding sites of the potential TF in the locus of the GOI using a combination of TFBIND, PROMO and MATCH

6. *In silico* validation of the predicted TF regulating expression of GOI

6.1. Checking GOI conservation: Extraction of the phylogenetic tree for the GOI from the Ensembl database (www.ensembl.org) by searching for the gene and then choosing Gene Tree from the Comparative Genomics section
Extraction of the alignment of the identified potential TF binding sites from the genomic alignments in the comparative genomics section on the Ensembl page of the GOI

6.2. Checking co-regulation of TF + GOI: Search NCBI GEO database for relevant datasets obtained from loss- or gain-of-function studies of the TF. Retrieval of the values for the potential TF and GOI via the GEO2R profile graph option.

6.3. Checking for TF binding: Downloading datasets of TF-specific ChIP-seq experiments in the public domain. After alignment and quantification, the resulting bam files can be used to analyse the promoter of GOI by utilizing the Integrative Genomics Viewer (IGV).

7. Experimental validation of the potential TF regulating expression of GOI

7.1. Testing direct interaction of the predicted TF with the GOI promoter

7.1.1. ChIP-qPCR: Perform chromatin immunoprecipitation using an antibody against the TF to test for capture of GOI promoter fragments.
Fix the desired cells or tissue (crosslink + quench) and incubate with the antibody against the TF. Test the retrieved DNA via PCR for presence of promoter sequences for the GOI.

7.1.2. Luciferase assays: Clone promoter fragment of the GOI upstream of luciferase. Transfect cells with the resulting vector and test functional influence of overexpression of the TF on luciferase activity. Influence of potential binding sites can be tested by mutating the original construct.

7.2. Testing co-regulation *in vitro/vivo*: Loss- and gain-of-function experiments for the predicted TF can further substantiate the finding. These experiments can include generation of or using available TF mutants/knock out lines of different model organisms or TF overexpression systems and testing for expression of GOI.

a

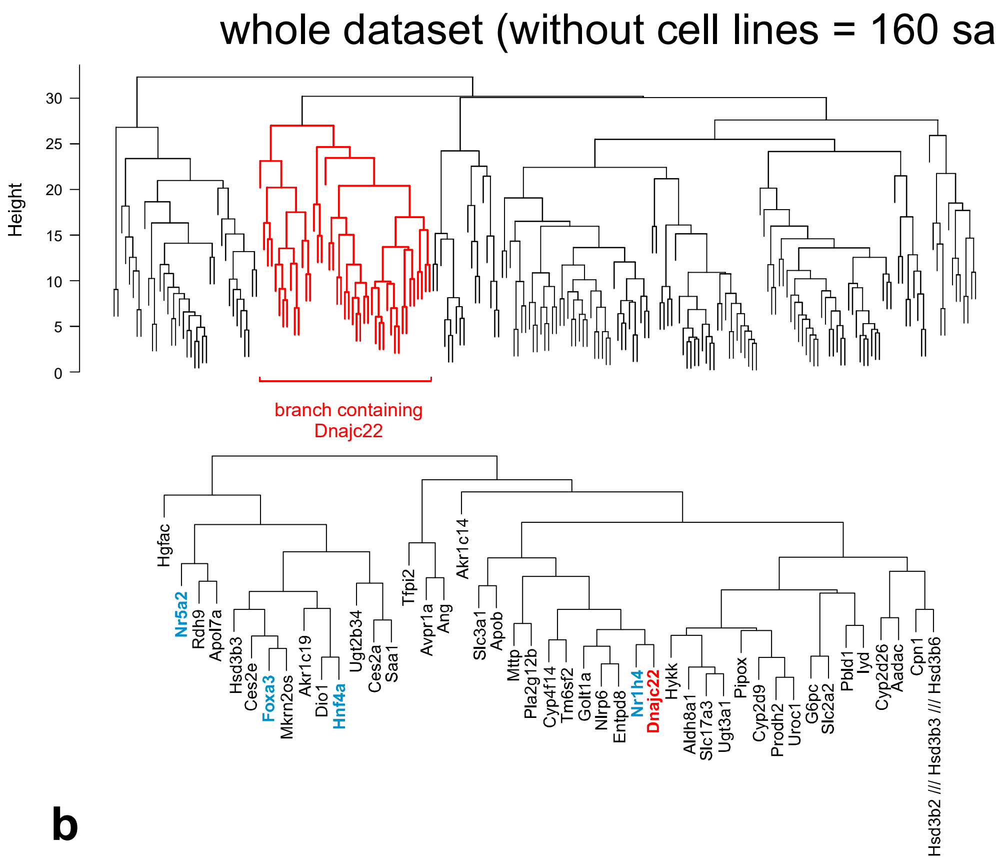

pcaGoPromoter

TFs	pvalue
Hnf1a	6.9e-14
Hnf1b	4.6e-09
Hnf4a	9.1e-09
Hnf4a	5.2e-07
Nr2f1	1.6e-05
Pparg	5.6e-05
Nr1h2	5.6e-05
Cebpa	2.2e-04
Tcf3	2.2e-04
Tcf7	7.9e-04
Prrx2	9.9e-04
Nfic	1.3e-03
Gata1	2.9e-03
Srf	3.8e-03
Rxra	3.8e-03
Dbx2	3.8e-03
Gata2	4.6e-03

iRegulon

TFs	NES	#Targets	#Motifs
Nr1h4	12.984	129	29
Hnf4a	4.845	112	10
Foxl1	4.691	70	25
Foxn4	4.199	17	1

b

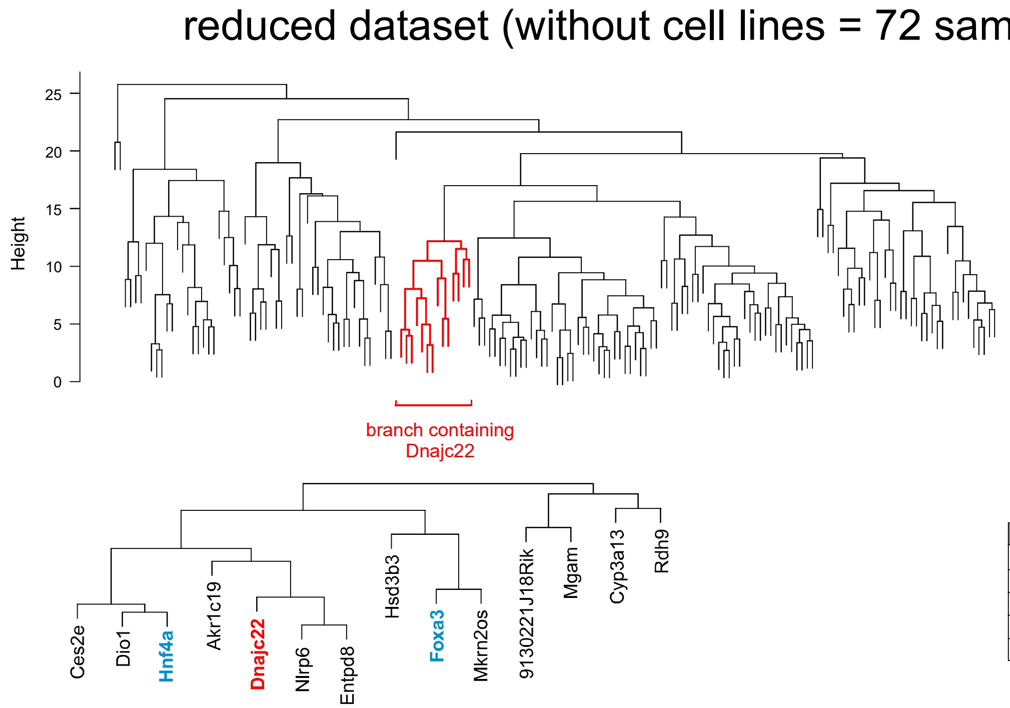

pcaGoPromoter

TFs	pvalue
Hnf4a	2.2e-05
Hnf1b	3.2e-04
Nr2f1	1.6e-03
Nr1h2	1.6e-03
Hnf4a	1.6e-03

iRegulon

TFs	NES	#Targets	#Motifs
Hnf4a	8.212	105	14
Nr1h4	6.397	81	12
Cdx1	5.691	45	5
Gata1	4.738	58	9
Zfp354c	4.326	34	3

Supplementary Figure 8 - SOM-clustering of murine data using different sample sizes

Hierarchical SOM-cluster for 225 genes (**a**) or 170 genes (**b**) co-regulated with murine Dnajc22 (highlighted in red). Transcription factors identified in the gene of interest-subcluster are marked in blue. Possible TFs for the genes contained in the SOM-cluster predicted by either iRegulon or pcaGoPromoter are depicted in the respective tables. TFs that are included in the SOM-cluster are marked in blue (TFs = transcription factors, NES = motif enrichment score, SOM = self-organizing map)
